# Short exposure to high salt in drinking solution leads to a cardiovascular phenotype of hypertension without changes in the blood volume of rats

**DOI:** 10.1113/EP090912

**Published:** 2023-01-30

**Authors:** Paula Magalhães Gomes, Julia Santos Batista, Renato Willian Martins Sá, Vagner Roberto Antunes

**Affiliations:** ^1^ Department of Physiology and Biophysics Institute of Biomedical Sciences University of Sao Paulo Sao Paulo SP Brazil

**Keywords:** blood volume, neurogenic hypertension, salt loading

## Abstract

Evidence suggests that hypertension induced by high salt intake is correlated with an autonomic imbalance that favours sympathetic hyperactivity and an increase in vascular resistance, indicating a neurogenic component to this pathology. Although there are several animal models in which to study salt‐induced hypertension with prolonged exposure to a high‐sodium diet, here we sought to investigate whether the increase in arterial blood pressure of rats subjected to a short exposure to high salt, with 2% NaCl drinking solution instead of water, relies on changes in the circulating blood volume. Male Wistar rats were divided randomly into three groups: euhydrated (EU, *n* = 10), salt loaded (SL, *n* = 13) and water deprived (WD, *n* = 6). The SL rats exhibited a significant increase in mean arterial blood pressure, with a large low‐frequency component of systolic arterial blood pressure variability, when compared with the EU group. Circulating blood volume did not differ between SL and EU rats, but it was lower in WD rats. Compared with EU rats, the [Na^+^] in cerebrospinal fluid was higher in SL rats and similar in magnitude to the WD rats. Plasma [Na^+^] did not differ between SL and EU rats, but it was higher in WD rats. Collectively, our data suggest that the hypertension induced by a short exposure to high salt intake closely resembles a neurogenic mechanism, but not a blood volume‐dependent mechanism, with cumulative [Na^+^] in the cerebrospinal fluid that could be associated with changes in the neurochemistry of autonomic nuclei, which are highly susceptible to osmotic stress related to high salt consumption.

## Abstract (highlights)

- **What is the central question of this study?**
- Is the cardiovascular phenotype of high blood pressure observed in rats salt loaded with 2% NaCl in drinking solution a blood volume‐dependent hypertension?
- **What is the main finding and its importance?**
- Animals exposed to 2% NaCl drinking solution develop hypertension, with dominance of sympathetic outflow and high [Na^+^] in the cerebrospinal fluid, but without changes in the blood volume. The phenotype of salt‐dependent hypertension might be related to accumulation of [Na^+^] in the cerebrospinal fluid, which makes it an interesting animal model in which to study the neuronal pathways involved in control of the circulation in osmotic challenge conditions.

## 1 INTRODUCTION

The current pattern of excessive salt consumption by the population is one of the main factors in the pathogenesis of primary hypertension (Elijovich et al., 2016). Although widely recognized in clinical practice as a trigger for hypertension, the mechanism by which increased salt intake leads to increased arterial blood pressure (ABP) is still poorly understood. In the 1970s, it was believed that hypertension resulting from sodium intake was derived from a primary renal dysfunction, because high sodium consumption would lead to water retention and consequent increase in circulating blood volume, which became known as the Guyton–Coleman model (Guyton et al., 1972). Several studies indicate that although the Guyton–Coleman model is very useful for understanding long‐term control mechanisms of ABP, it underestimates the role of the brain and the autonomic nervous system (ANS) in neural control of the circulation, now called neurogenic hypertension (Colombari et al., 2001; Guyenet, 2006; Guyenet et al., 1989, 2020).

Significant scientific advances in our understanding of the pathophysiological mechanisms of salt‐dependent hypertension were made possible by the use of experimental animal models, mainly in rodents (Lerman et al., 2019). Dahl and colleagues made a significant progress in this field by using an inbred strain of rats, called Dahl salt‐sensitive rats, which when fed a high‐salt diet (8% NaCl) rapidly develop hypertension with progressive renal injury (Dahl et al., 1962). They concluded that increased intake of high‐salt diets results in increased osmolality and plasma sodium content, leading to increased blood volume and progressing to hypertension (Dahl et al., 1962, 1968). In contrast, many others provided evidence for a role of neurogenic factors in the genesis of hypertension, characterized by an imbalance in the autonomic control of the circulation favouring hyperactivity of the sympathetic nervous system and, consequently, an increase in peripheral vascular resistance (Adams et al., 2007; Antunes et al., 2006; Guyenet, 2006; Stocker et al., 2010).

Recently, we and others have adopted a very useful experimental animal model of salt‐induced neurogenic hypertension, which consists of animals provided with 2% NaCl solution instead of drinking water (Choe et al., 2015; Ribeiro et al., 2020, 2015). Previously, we have shown that genesis and maintenance of high ABP in this animal model involved significant participation of the ANS, evidenced by an increase in sympathetic nerve activity (Ribeiro et al., 2015). However, we have been targeted by the criticism that this experimental model of salt‐loaded rats would more closely resemble a model of dehydration and that the cardiovascular phenotype of hypertension regularly observed in this model could be a consequence of changes in blood volume, rather than the involvement of a neurogenic component. Hence, we aimed to determine, in non‐anaesthetized rats, whether the increase in ABP induced by a high salt intake, owing to 2% NaCl in the drinking solution for 7 days, is a blood volume‐dependent hypertension.

## 2 METHODS

### 2.1 Ethical approval

All experimental procedures were approved by the Ethical Committee for Animal Research of the Institute of Biomedical Sciences, University of São Paulo (#7446300120 and #1111082011), and were conducted in accordance with the Ethical Principles in Animal Research mandated by the Brazilian College of Animal Experimentation and conformed to the regulations described in the editorial by Grundy (2015). Male Wistar rats (*n* = 29) weighing 290–400 g were obtained from the Animal Facility Centre at University of São Paulo and housed at a constant temperature of 22−24°C and a humidity of 50–60%, under a controlled light–dark cycle (12 h–12 h) with a regular rat chow diet (Nuvilab, Paulínia, SP, Brazil). The animals were divided randomly into three groups: euhydrated rats (EU group, *n* = 10) provided with tap water ad libitum; salt‐loaded rats (SL group, *n* = 13) that received 2% NaCl as the drinking solution ad libitum instead of tap water for 7 days; and water‐deprived rats (WD group, *n* = 6) that were housed without water, but with food, for 48 h.

### 2.2 Fluid consumption and urine output

**FIGURE 1:**
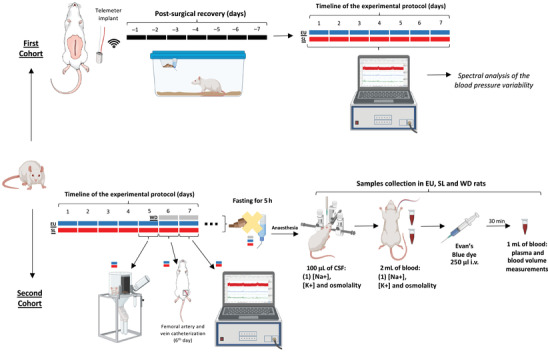
Schematic representation of the experimental design and time line of the protocol. Details are given in the Methods section.

In order to monitor fluid ingestion and excretion, EU and SL rats were housed individually in metabolic cages (Tecniplast) for a single period of 48 h and provided with tap water or 2% NaCl solution, respectively, and regular chow ad libitum. The volume of urine excreted and the intake of water or 2% NaCl solution were measured gravimetrically during 24 h in the metabolic cage (Figure 1). Urine was centrifuged and frozen at −20°C for further analysis of electrolytes.

### 2.3 Monitoring of cardiovascular parameters in non‐anaesthetized rats

All surgical procedures were performed in animals anaesthetized with 2–5% isoflurane in 100% O_2_ air (Isoforine; Cristália, Produtos Químicos‐Farmacêuticos, SP, Brazil), and the level of anaesthesia was checked frequently by assessing limb withdrawal reflexes to noxious pinching. The cardiovascular parameters were measured in two distinct cohorts of EU and SL rats using two different technical procedures (Figure 1), as follows.

#### 2.3.1 Radiotelemetry system

Systolic blood pressure (SBP), diastolic blood pressure, mean arterial pressure (MAP) and heart rate were recorded with a telemetry system (Kaha Sciences, Houston, TX, USA) in EU (*n* = 5) and SL (*n* = 7) animals, as previously described by researchers in our laboratory (Ribeiro et al., 2020). Briefly, the telemeters were implanted ≥7 days before recording of cardiovascular parameters began. A mid‐line laparotomy was made in the supine position, and the intestines were reflected to expose the abdominal aorta. The tip of the catheter connected to a transmitter (TRM54P) was inserted into the abdominal aorta caudal to the root of the left renal artery and held in place with tissue adhesive (Vetbond; 3M). The body of the transmitter was sutured to the ventral wall of the abdominal cavity.

#### 2.3.2 Catheterization of blood vessels

The femoral artery and vein were catheterized in a second cohort of EU (*n* = 4) and SL (*n* = 6) animals, which were subjected to the metabolic cage and blood volume measurements. Briefly, polyethylene catheters (PE‐10 connected to PE‐50; Clay Adams, Parsippany, NJ, USA) were inserted into the femoral artery and vein for MAP and heart rate monitoring and for dye injections, respectively. The cardiovascular parameters were recorded 24 h after surgery at 1000 Hz sampling rate and acquired in the software LabChart v.8.1.5 (ADInstruments, Australia). After all surgical interventions, animals received, once a day, antibiotic (Penikel LA; penicillin G procaine 15,000,000 IU; Ceva Animal Health, Paulínia, SP, Brazil) and ketoprofen (Biofen 1%; Biofarma, Brazil) subcutaneously, until the day of data collection.

### 2.4 Spectral analysis of blood pressure variability

The sympathetic modulation on blood pressure was estimated by spectral analysis of SBP variability in the radiotelemetered group (Figure 1). Sequences of consecutive SBP values were taken from recording segments and analysed by Cardioseries Program (v.2.2; SP, Brazil) as previously performed by Ribeiro et al. (2020).

### 2.5 Collection of cerebrospinal fluid and blood and measurements of osmolality, Na^+^ and K^+^

In order to standardize the body fluid balance among groups the collections of cerebrospinal fluid (CSF) and blood samples were performed in all groups of animals deprived of food and liquid for 5 h (EU *n* = 4 or 5, SL *n* = 6 and WD *n* = 6; Figure 1). The CSF and blood samples were collected in animals under general anaesthesia, as previously described (de Souza et al., 2021; Gomes et al., 2017). Briefly, animals were placed in a stereotaxic apparatus, and the occipital muscles were removed to reach the atlanto‐occipital membrane. A 30‐gauge needle was inserted into the cisterna magna, and 100 μl of clean CSF was collected (Figure 1). Afterwards, a blood sample was taken, and haematocrit was measured immediately using heparinized capillaries and a centrifuge (Hettich 2076 Rotor for Haematocrit, Germany) 1,000 *g* for 10 min. Plasma and CSF osmolalities were measured with a vapour pressure osmometer (Vapro 5600; Wescor, Logan, UT, USA), and the [Na^+^] and [K^+^] were measured using flame photometry (MicroNal B462; Tecnal, Piracicaba, SP, Brasil). The analyses of CSF were performed in duplicate, and for the plasma samples in triplicate.

### 2.6 Plasma and blood volume measurements: Evan's Blue protocol

On day 7 of 2% NaCl solution (SL) and water (EU) intake, and on day 2 of water deprivation (WD), the animals, under general anaesthesia, received an injection of Evan's Blue dye (0.584 mg/ml; 250 μl) through the femoral vein catheter, followed by a flush of 100 μl of 0.9% NaCl. After 30 min, 1 ml of blood was sampled from the femoral artery catheter (Figure 1). The Evan's Blue protocol has been described elsewhere (de Souza et al., 2021; Radu & Chernoff, 2013), but briefly, the concentration of Evan's Blue in the plasma was determined spectrophotometrically (Femto 6000 Plus) at a wavelength of 620 nm against a plasma blank from an untreated animal. The volumes injected or withdrawn were considered in the final accounts of circulating volume. At the end of sample collection, animals were killed by exsanguination.

### 2.7 Statistical analysis

Results are reported as the mean ± SD. Comparisons between groups were performed by Student's unpaired *t*‐test, one‐way or two‐way ANOVA for repeated measures, when applicable. Bonferroni's post‐hoc test or the Holm–Sidak post‐hoc test was used for multiple comparisons after ANOVA. All data were analysed using GraphPad Prism v.9.0.2 software for Windows (GraphPad Software, San Diego, CA, USA). Differences between pairs of means were considered significant when the probability of type I error was <5% (*P* < 0.05).

## 3 RESULTS

### 3.1 Intake of 2% NaCl solution causes a rapid and progressive increase in the ABP of rats

**FIGURE 2:**
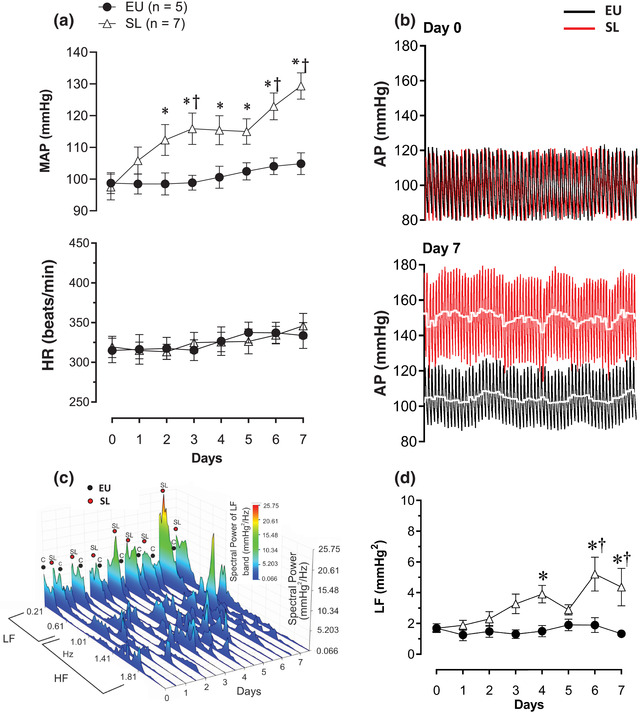
(a) Time course of the increase in the mean arterial pressure (MAP; in millimetres of mercury) and no change in the heart rate (HR; in beats per minute) of rats exposed to high salt intake (2% NaCl) for 7 days (SL group, *n* = 7) compared with the euhydrated (EU) group (water only, *n* = 5) measured by the radiotelemetry system. (b) Representative traces of change in the arterial pressure (AP; in millimetres of mercury) of one animal in the SL group (red) and one in the EU group (black) at the start (day 0) and after 7 days of high‐salt exposure. (c,d) Spectral power bands of the systolic blood pressure (SBP; in millimetres of mercury squared per herz; c) and area under curve of the low‐frequency (LF) band (d), showing temporal profile of the incremental influence of sympathetic activity on SBP regulation in SL rats during 7 days of high‐salt exposure. ^*^ *P* < 0.01 compared with day 0; ^†^ *P* < 0.01 compared with EU. Two‐way ANOVA followed by the Holm–Sidak post‐hoc test.

The progression of hypertension in SL rats was measured using a radiotelemetry system. The MAP increased progressively from day 2 to 7. Figure 2a,b shows the maximal difference in MAP observed when comparing day 7 versus day 0 in the same group [MAP: 97 ± 4 mmHg (day 0, SL) vs. 129 ± 4 mmHg (day 7, SL), *P* < 0.0001]. Compared with EU rats, the maximal difference was also observed on day 7 [MAP: 104 ± 3 mmHg (EU, day 7) vs. 129 ± 4 mmHg (SL, day 7), *P* < 0.0001]. In the SL group, MAP was higher on day 4 compared with day 0 [MAP: 97 ± 4 mmHg (SL, day 0) vs. 115 ± 3 mmHg (SL, day 4), *P* = 0.1038]. No significant changes in the heart rate were observed between EU and SL groups, Figure 2a. The overlaid bood pressure traces shown in Figure 2b were taken from single representative EU and SL rats during day 7 postintervention.

### 3.2 Spectral analysis of SBP variability shows a major influence of sympathetic activity in salt‐loaded rats

The SBP spectra shown in Figure 2c show the power of the low‐frequency (LF) band increasing progressively from day 0 to 7 in SL rats, reflecting an incremental influence of sympathetic activity on SBP regulation. Significant differences were observed on days 4, 6 and 7 compared with day 0 (Figure 2d). The maximal difference occurred on day 6 compared with day 0 in the same group [LF: 1.6 ± 0.2 mmHg^2^ (day 0, SL) vs. 5.2 ± 1 mmHg^2^ (day 6, SL), *P* < 0.0001]. Comparing LF spectral power of the SL group with the EU group, significant differences were observed on days 6 and 7 [LF day 6: 1.8 ± 0.4 mmHg^2^ (EU) vs. 5.2 ± 1 mmHg^2^ (SL), *P* < 0.0001] and 7 [LF day 7: 1.3 ± 0.2 mmHg^2^ (EU) vs. 4.3 ± 1.2 mmHg^2^ (SL), *P* < 0.0001].

### 3.3 Intake of 2% NaCl solution leads to accumulation of Na^+^ in the CSF, but not in the plasma

**FIGURE 3:**
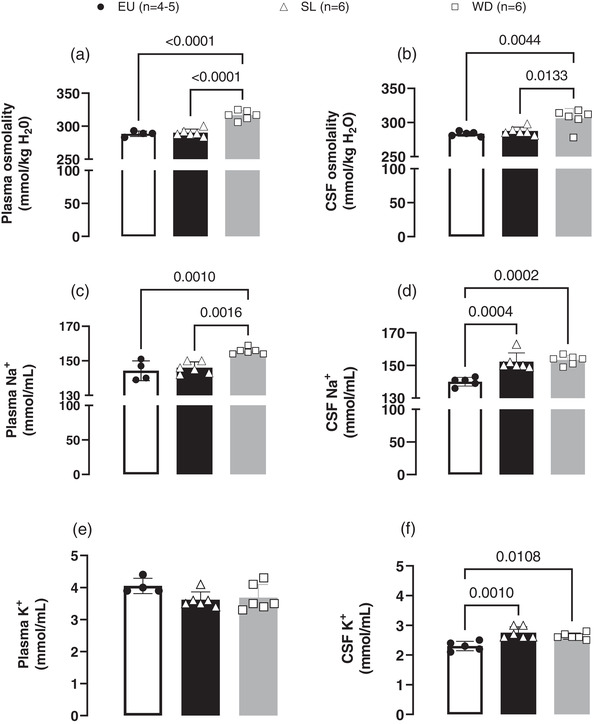
Plasma and cerebrospinal fluid (CSF) [Na^+^], [K^+^] and osmolality. (a,b) Osmolality measured in fresh samples in plasma and CSF, respectively, (c,d) [Na^+^] in the plasma and CSF, respectively. (e,f) [K^+^] in plasma and CSF, respectively. Scattered circles (EU, *n* = 4 or 5), triangles (SL, *n* = 6) and squares (water‐deprived, WD, *n* = 5) represent individual values, and bars represent mean values with the respective SD for each group. One‐way ANOVA for repeated measurements followed by Bonferroni's post‐hoc test; differences between pairs of means are indicated by *P*‐values.

Fluid consumption, urine volume and ABP were higher in SL compared with EU rats (Table 1). Figure 3a,b shows that plasma and CSF osmolality did not differ between EU and SL rats, but were higher in the WD rats [plasma (EU: 288 ± 4 mosmol/kg H_2_O vs. SL: 290 ± 5 mosmol/kg H_2_O vs. WD: 316 ± 7 mosmol/kg H_2_O); CSF (EU: 283 ± 3 mosmol/kg H_2_O vs. SL: 287 ± 5 mosmol/kg H_2_O vs. WD: 306 ± 10 mosmol/kg H_2_O]. Basal [Na^+^] in the plasma did not differ between EU (144 ± 6 mmol/ml) and SL rats (146 ± 3 mmol/ml), but it was higher in WD rats (155 ± 2 mmol/ml; Figure 3c). No significant change in the plasma [K^+^] was observed among groups (Figure 3e).

**TABLE 1:** Fluid consumption and urine output.

Parameter	Euhydrated group (*n* = 4)	Salt‐loaded group (*n* = 6)
Water or 2% NaCl intake (ml/24 h)	26.6 ± 2.3	120.2 ± 59.9*
Urine output (ml/24 h)	18.2 ± 4	86 ± 20.5*
Mean arterial pressure (mmHg)	109 ± 1	124 ± 6*
Systolic blood pressure (mmHg)	127 ± 1	141 ± 8*
Diastolic blood pressure (mmHg)	95 ± 2	110 ± 9*
Heart rate (beats/min)	378 ± 31	373 ± 32

*Note*: Measurements for 24 h of fluid consumption and urine output in the metabolic cage were performed in the second cohort between the day 5 and 6 of the protocol and blood pressures were monitored on day 7 of high salt exposure (2% NaCl) in SL (*n* = 6) and water in EU (*n* = 4) rats.

* Significantly different from the EU group (Student's unpaired *t*‐test).

Unlike plasma, the [Na^+^] in CSF was higher in SL rats (152 ± 5 mmol/ml), similar to the increase observed in WD rats (153 ± 3 mmol/ml), when compared with EU rats (140 ± 3 mmol/ml; Figure 3d). Figure 3e shows that the [K^+^] in the CSF of SL rats was higher (2.7 ± 0.1 mmol/ml) in comparison to EU rats (2.3 ± 0.1 mmol/ml) and WD rats 2.6 ± 0.1 mmol/ml).

### 3.4 Intake of 2% NaCl solution does not affect the circulating blood volume

**FIGURE 4:**
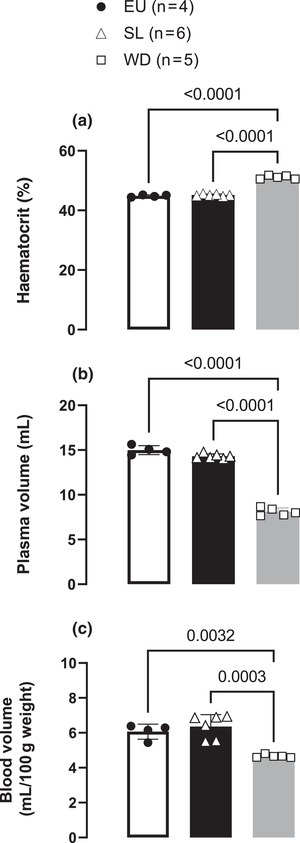
Haematocrit (a), plasma volume (b) and circulating blood volume per 100 g body weight (c) in euhydrated (EU), salt‐loaded (SL) and water‐deprived (WD) rats. Scattered circles (EU, *n* = 4 or 5), triangles (SL, *n* = 6) and squares (WD, *n* = 5) represent individual values, and bars represent mean values with the respective SD for each group. One‐way ANOVA for repeated measurements followed by Bonferroni's post‐test; differences between pairs of means are indicated by *P*‐values.

Figure 4a shows that haematocrit did not differ between EU and SL rats [EU: 44.8 ± 0.3% vs. SL: 45.1 ± 0.3%), but it was higher in WD rats (51.1 ± 0.6%). High salt intake did not change the plasma volume (SL: 14.4 ± 0.3 ml) and circulating blood volume (SL: 6.3 ± 0.6 ml) when compared with EU rats (15.1 ± 0.5 ml and 6.1 ± 0.4 ml, respectively; Figure 4b,c). For comparison, we analysed the same parameters in rats deprived of water for 48 h and, as expected, the plasma volume (WD: 8.1 ± 0.4 ml) and blood volume (WD: 4.6 ± 0.1 ml) were lower when compared with EU and SL rats.

## 4 DISCUSSION

The present study demonstrated that rats restricted to drinking 2% NaCl solution instead of tap water for 7 days develop a progressive and sustained increase in ABP, with an evident autonomic influence identified by an increase in the LF component of the SBP variability, without a change in circulating blood volume, but with significant accumulation of [Na^+^] in the CSF.

Several animal models have been used to mimic, as closely as possible, the human pathology of salt‐dependent hypertension. The most classic is the Dahl‐salt sensitive rat, a genetic rat model, wherein animals fed with a high content of salt in the diet (8% NaCl) and with water ad libitum are prone to develop hypertension with progressive renal injury (Dahl et al., 1962). Likewise, many other studies have shown that different strains of rats exposed to a high‐sodium diet and with water ad libitum [e.g., Wistar rats fed with 2% NaCl in the diet for 12 weeks after weaning (Gomes et al., 2017) and adult Sprague–Dawley rats fed for 10 days with 8% dietary NaCl (Gu et al., 2008; Osborn et al., 1993)] develop and increased ABP, with significant sympathetic responsiveness, as part of salt‐sensitive hypertension.

Here, we used an experimental rat model of salt loading with 2% NaCl in drinking solution instead of water, in which the animals develop a cardiovascular phenotype of elevated ABP during a short duration of exposure to the high‐salt conditions. This is a practical animal model used to study the neural control of circulation; however, it has been criticized by our peers, who suggest that this animal model resembles a dehydration model, with possible changes in the blood volume, and that the hypertensive phenotype observed would not be essentially neurogenic in origin, with sympathetic hypertonia, as we have previously shown (Ribeiro et al., 2020, 2015). In order to bridge this gap, we decided to evaluate the circulating blood volume and plasma volume, in addition to plasma and CSF osmolality and [Na^+^] as an index of changes in the hydroelectrolytic balance, and our data show that volaemia is not changed in SL rats, nor are the plasma [Na^+^] and osmolality, but the [Na^+^] in the CSF is higher in SL rats.

Although the physiological mechanisms by which a short exposure of an animal to high salt intake leads to an increase in ABP are not fully elucidated, there is strong evidence for a neurogenic component. Supporting this assertion, here we have shown that the LF component of SBP variability, an index of the sympathetic influence on the circulation, is higher in SL when compared with EU rats. Corroborating this hypothesis, we have previously shown that rats exposed to 2% NaCl in the drinking solution exhibited a significant increase in lumbar sympathetic nerve activity and high ABP, with the involvement of vasopressin V1a receptor activation in the paraventricular nucleus of the hypothalamus in these responses (Ribeiro et al., 2015). Kim et al. (2011), using the same experimental model of salt loading, showed that GABAergic responses of magnocellular vasopressin‐secreting neurones in the paraventricular nucleus switched between inhibition and excitation when rats were exposed to 7 days of salt loading, a mechanism contributing to the development of neurogenic hypertension in this rat model. More recently, a study from our laboratory reinforced the hypothesis that the hypertension induced by salt overload for 7 days is of neurogenic origin, because ablation of catecholaminergic neurons from the rostral ventrolateral medulla attenuated the hypertension and the sympathetic influence on the neural control of the circulation in salt‐loading conditions (Ribeiro et al., 2020). Collectively, these findings suggest that the hypertension induced by salt loading in rats exposed to 2% NaCl for 7 days is essentially caused by a neurogenic component, but not by changes in the blood volume in this experimental model.

One of the interesting findings observed in our salt‐loading rat model was the increase in the [Na^+^] in the CSF, without a significant change of this ion in the plasma or a change in the osmolality. Although an increase in [Na^+^] in the CSF would be expected to cause a direct increase in its osmolality, recent studies have reported that osmolality and [Na^+^] in different extracellular fluids of high salt‐fed rats might change differentially (Nikpey et al., 2017). Thowsen et al. (2022) identified that periods of hyperosmotic stress led to storage of Na^+^ in the skin and the cardiac and smooth musculature, being ‘inactivated’ from the point of view of the osmotic gradient, keeping the plasma in normal conditions. Elevated [Na^+^] in the CSF has been identified in another model of salt‐sensitive hypertension with moderate sodium consumption, without a change in the plasma (Gomes et al., 2017). These data suggest a strong relationship between high sodium consumption and its accumulation in the CSF, with the genesis and maintenance of high ABP.

Clinical studies that have correlated human hypertension and CSF composition are limited, but some of them indicate that the CSF and plasma [Na^+^] in hypertensive humans are not the same as in normotensive subjects. Kawano et al. (1992) demonstrated that in patients subjected to a high salt intake for 7 days (16–18 g/day), ABP and the [Na^+^] in CSF and serum were significantly higher in the high‐salt period than in the low‐salt period. More recently, Souza et al. (2020) found a high sodium content in the CSF of hypertensive patients compared with normotensive subjects, without significant changes of this ion in the plasma or of plasma and CSF osmolality between groups, suggesting that there is a clinical significance of high CSF [Na^+^], which might play a key role in the pathogenesis of human hypertension.

It is important to emphasize that we do not advocate that this model of salt loading is a model of salt‐sensitive hypertension, but rather that it is an interesting model to study the potential neuronal pathways of autonomic brain nuclei involved in the neural control of the circulation in conditions of an osmotic challenge. Additionally, this rat model of salt loading does not seem to resemble a dehydration model, at least not within a physiological range that would be sufficient to alter haemodynamic conditions to cause volume‐dependent hypertension.

In conclusion, our data suggest that the rat model of salt loading with 2% NaCl in drinking solution instead of tap water for 7 days is a neurogenic hypertension model, without any changes in plasma volume or circulating blood volume, with sympathetic hypertonia (Ribeiro et al., 2015), but mainly with accumulation of sodium in the CSF that could be associated with changes in the neurochemistry of autonomic nuclei, which are highly susceptible to osmotic stress related to high salt consumption.

### 4.1 Limitations

One possible caveat of this study is that all experiments were performed in male Wistar rats, and we cannot say that females would exhibit the same pattern of cardiovascular phenotype when subjected to 2% NaCl solution intake. The risk factors for cardiovascular diseases such as hypertension are multifactorial, but it is known there are pathophysiological characteristics that differ between the sexes (Bayorh et al., 2001), which should be investigated.

### 4.2 Perspectives

The hypertensive phenotype observed in this rat model of short exposure to high salt intake could be related to the high [Na^+^] in the CSF and to the sympathetic hypertonia and the increase in ABP. However, the physiological mechanisms are not clear, and it is not known whether intervention in the [Na^+^] in the CSF would change ABP during an established hypertensive state. In addition, we do not know how the choroid plexus epithelial cells deal with the sodium ions actively transported into the brain ventricles to keep the osmotic gradient in an environment of CSF hypernatraemia and hyperkalaemia, or the consequences on the neurotransmission of surrounding neuronal cells involved in neural control of the circulation. More studies are needed to understand the physiological mechanisms of the neuronal pathways involved in the salt‐induced hypertension, and this experimental animal model seems to be very useful for this purpose.

## AUTHOR CONTRIBUTIONS

Paula Magalhães Gomes designed and performed experimental protocols, analysed and interpreted the results, prepared the figures, drafted the manuscript and approved the final version. Julia Santos Batista and Renato Willian Martins Sá performed, analysed and interpreted the experiments and approved the final version of the manuscript. Vagner Roberto Antunes conceived and designed the research, interpreted results of experiments, edited and revised the manuscript and approved the final version. All authors agree to be accountable for all aspects of the work in ensuring that questions related to the accuracy or integrity of any part of the work are appropriately investigated and resolved. All persons designated as authors qualify for authorship, and all those who qualify for authorship are listed.

## CONFLICT OF INTEREST

None declared.

## Supporting information

Statistical Summary Document

## Data Availability

The data that support the findings of the present study are available from the corresponding author upon request. The data are openly available online in our GitHub repository (https://github.com/gomespm/EP_MS_NaCl_ShortExp.git).
